# Supplementary material for: The inhibitory action of the chaperone BRICHOS against the α-Synuclein secondary nucleation pathway
Source: Nat Commun. 2024 Nov 20;15:10038. doi: 10.1038/s41467-024-54212-2 (PMC11579453; doi:10.1038/s41467-024-54212-2)
Supplement: Supplementary file 1 — Supplementary Information [file 41467_2024_54212_MOESM1_ESM.pdf]

The inhibitory action of the chaperone BRICHOS against α-Synuclein secondary nucleation pathway

Dhiman Ghosh[1#], Felix Torres[1#], Matthias M. Schneider[2], Dzmitry Ashkinadze[1], Harindranath Kadavath[1,3],Yanick Fleischmann[1], Simon Mergenthal[4], Peter Güntert[1,5], Georg Krainer[2], Ewa A. Andrzejewska[2], Lily Lin[2], Jiapeng Wei[2], Enrico Klotzsch[4*], Tuomas Knowles[2,6*] and Roland Riek[1]*

# equally contributing authors

[1] Laboratory of Physical Chemistry, ETH Zürich, Vladimir-Prelog-Weg 2, CH-8093 Zürich, Switzerland,

[2]Department of Chemistry, University of Cambridge, Lensfield Road, Cambridge CB2 1EW, U.K

[3] St. Jude Children's Research Hospital, Memphis, Tennessee, United States
[4]Institute for Biology, Experimental Biophysics / Mechanobiology, Humboldt-Universität zu Berlin, 10115 Berlin, Germany

[5]Institute of Biophysical Chemistry, Center for Biomolecular Magnetic Resonance,

Goethe University Frankfurt am Main, 60438 Frankfurt am Main, Germany

[6]Cavendish Laboratory, Department of Physics, University of Cambridge, JJ Thomson Avenue, Cambridge CB3 0HE, U.K.

* to whom correspondence should be addressed to: roland.riek@phys.chem.ethz.ch, enrico.klotzsch@hu-berlin.de, tpjk2@cam.ac.uk

The file includes:

Supplementary figures 1 to 11 Supplementary Table 1

Materials and Methods

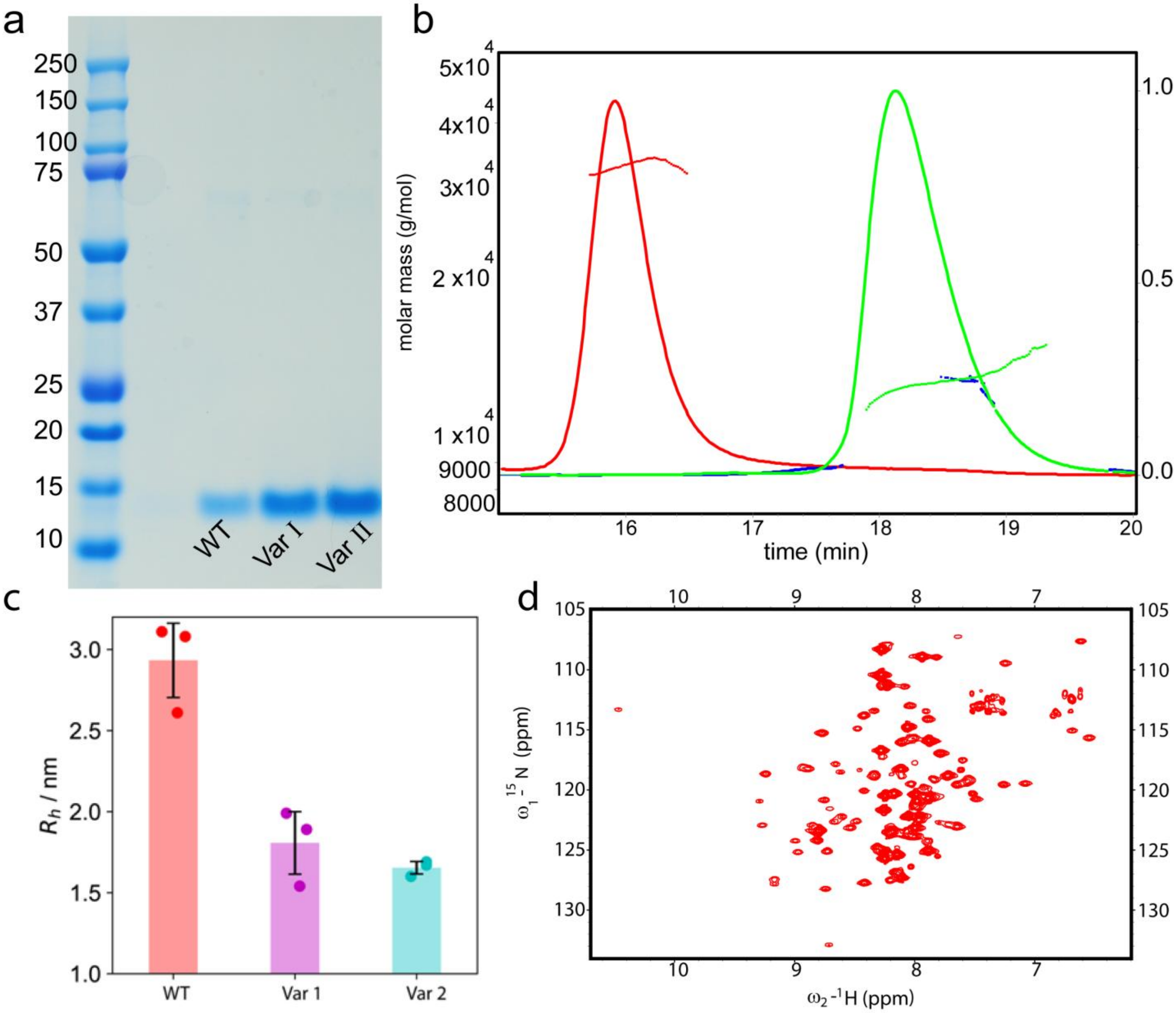

Supplementary Figure 1. Biophysical characterization of WT proSP-C BRICHOS and its variants. (a) SDS-PAGE images of WT proSP-C BRICHOS and its two artificial variants I and II showing presence of a single band at ~ 12 kDa. Although WT proSP-C BRICHOS has been shown to form trimer, in presence of SDS it denatures into monomer. (**b**) Multiangle light scattering of WT proSP-C BRICHOS (red) and variant II (green) shows different elution profile, which further confirmed the formation of trimer by WT and monomer by variant II. (**c**) Microfluidic Diffusional Sizing showing higher hydrodynamic radius of the WT proSP-C BRICHOS compared to its corresponding variants, which further calculated to be a trimer for WT proSP-C BRICHOS and monomeric for the variants. 3 independent experiments were performed and the corresponding S.D is presented here. (**d**) [^{15}N,^{1}H]-TROSY spectrum of WT proSP-C BRICHOS at 298 K.

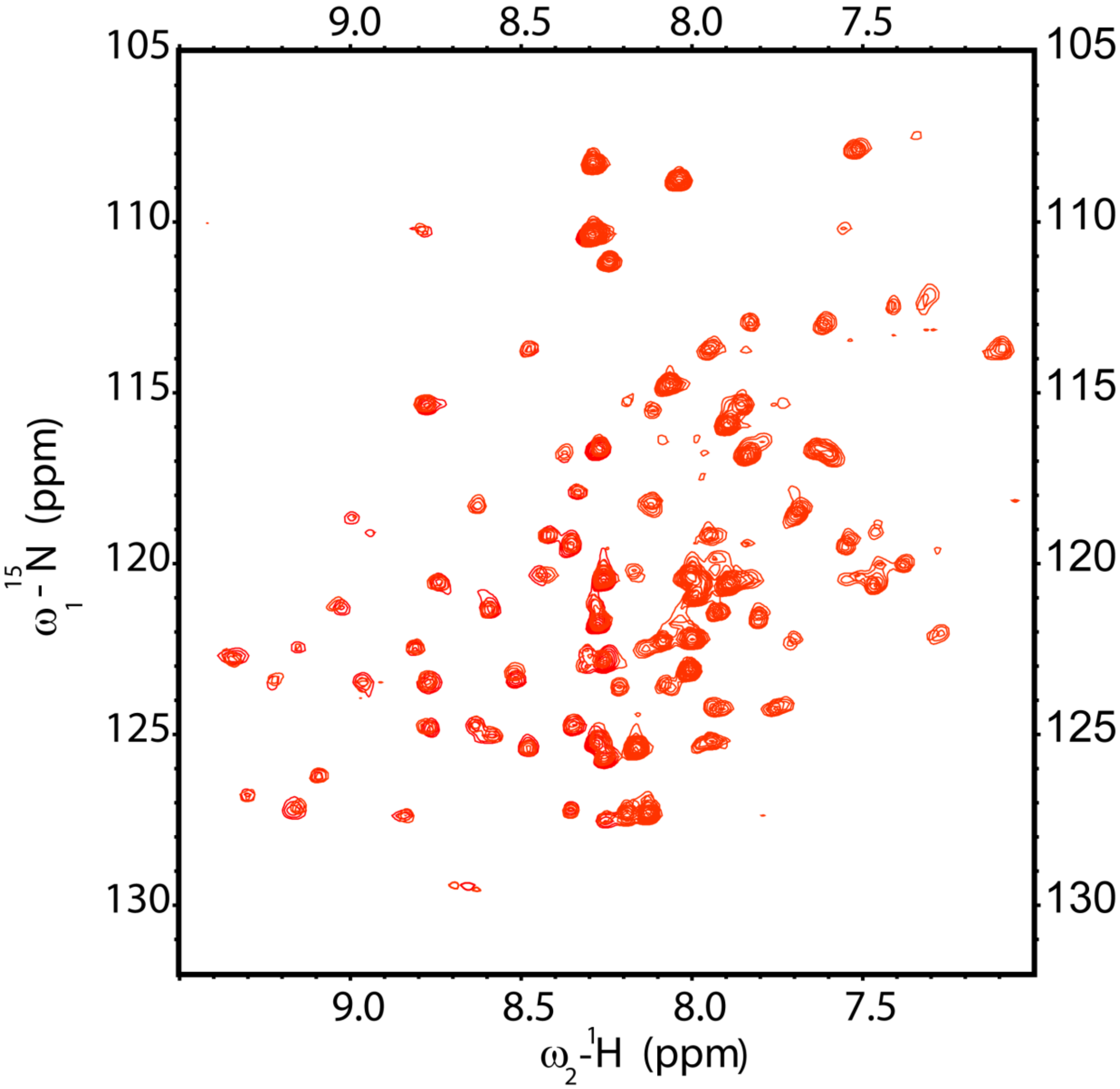

Supplementary Figure 2. [^{15}N,^{1}H]- TROSY spectrum of ^{15}N-labeled proSP-C BRICHOS Var II. [^{15}N,^{1}H]- TROSY spectrum of ^{15}N-labeled proSP-C BRICHOS Var II was recorded at pH ~6.8, phosphate buffer, 303 K on 700 MHz ^{1}H frequency.

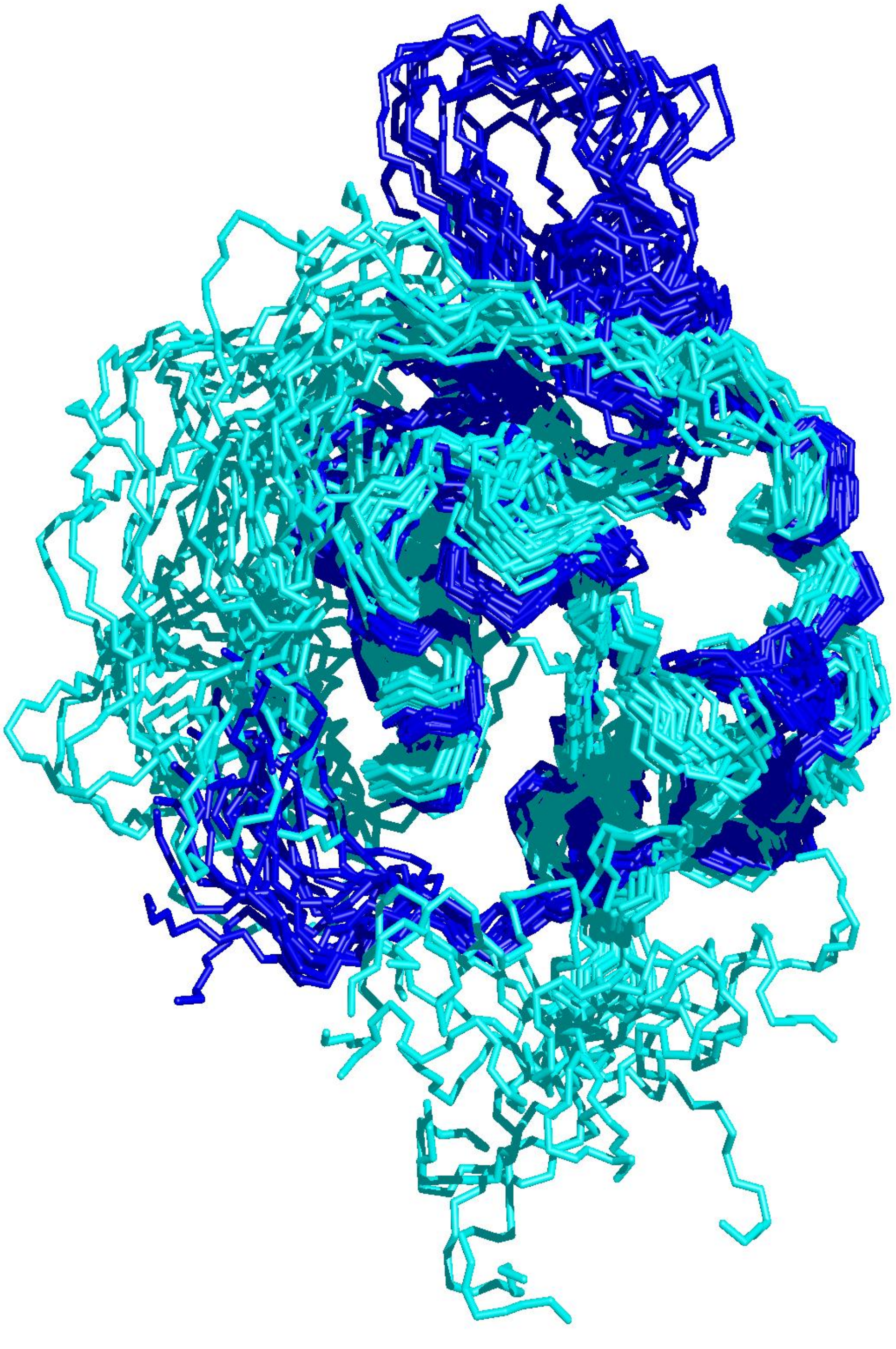

Supplementary Figure 3. Overlay structure of trimeric and monomeric BRICHOS. Overlay structure of proSP-C BRICHOS Trimer (blue) and proSP-C BRICHOS Var II (cyan), which has been shown to form a monomer.

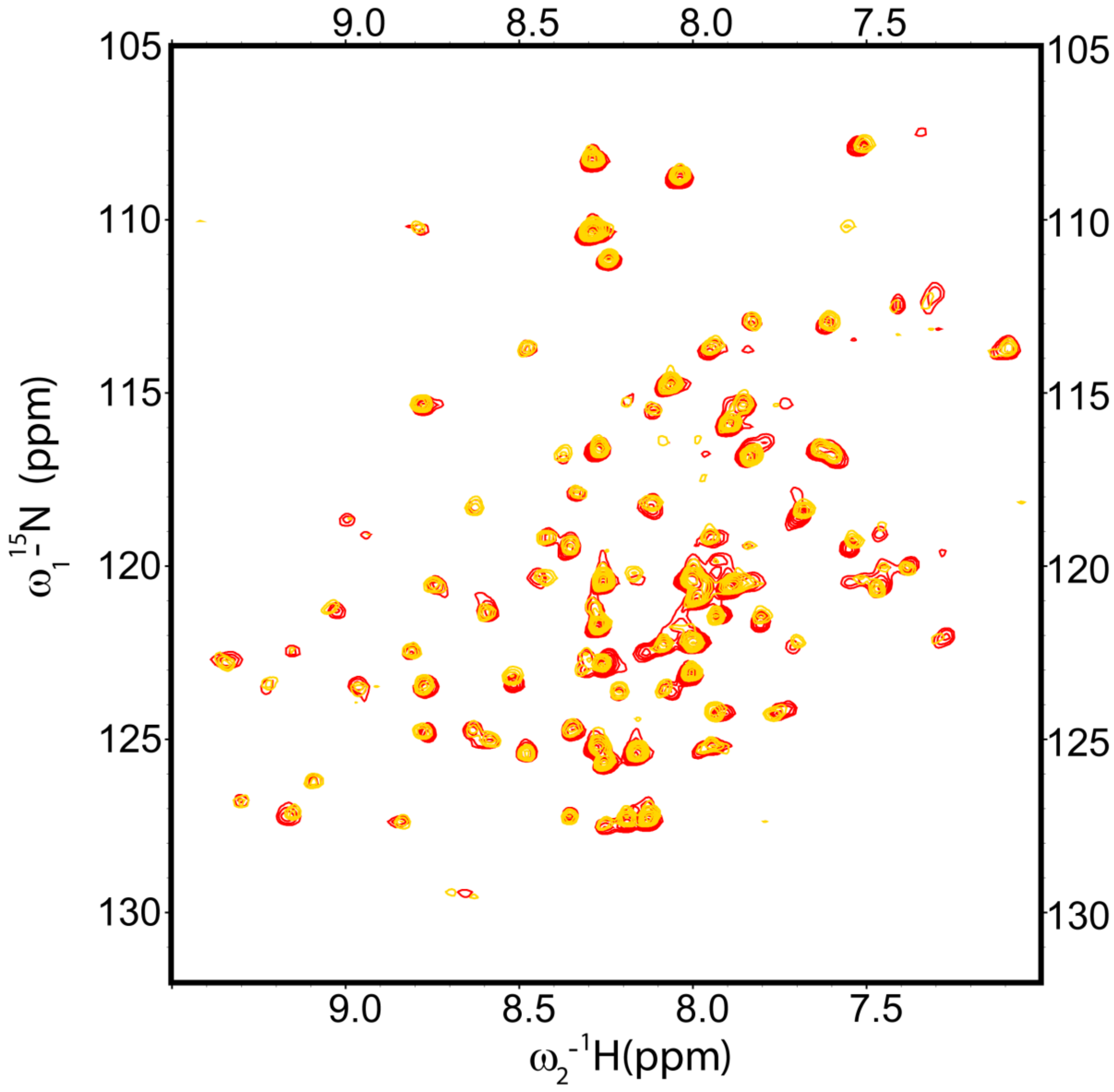

Supplementary Figure 4. Client peptide A does not interact with proSP-C BRICHOS Var II domain as revealed by NMR. [^{15}N,^{1}H]-TROSY spectra of 100 µM ^{15}N-labeled proSP-C BRICHOS Var II in phosphate buffer, pH 6.8 at 303 K in absence (red) and presence (orange) of client peptide A (KKVVVVVVKK) at a concentration of 1 mM. The near perfect superposition of the two spectra indicates lack of binding between proSP-C BRICHOS Var II domain and peptide A.

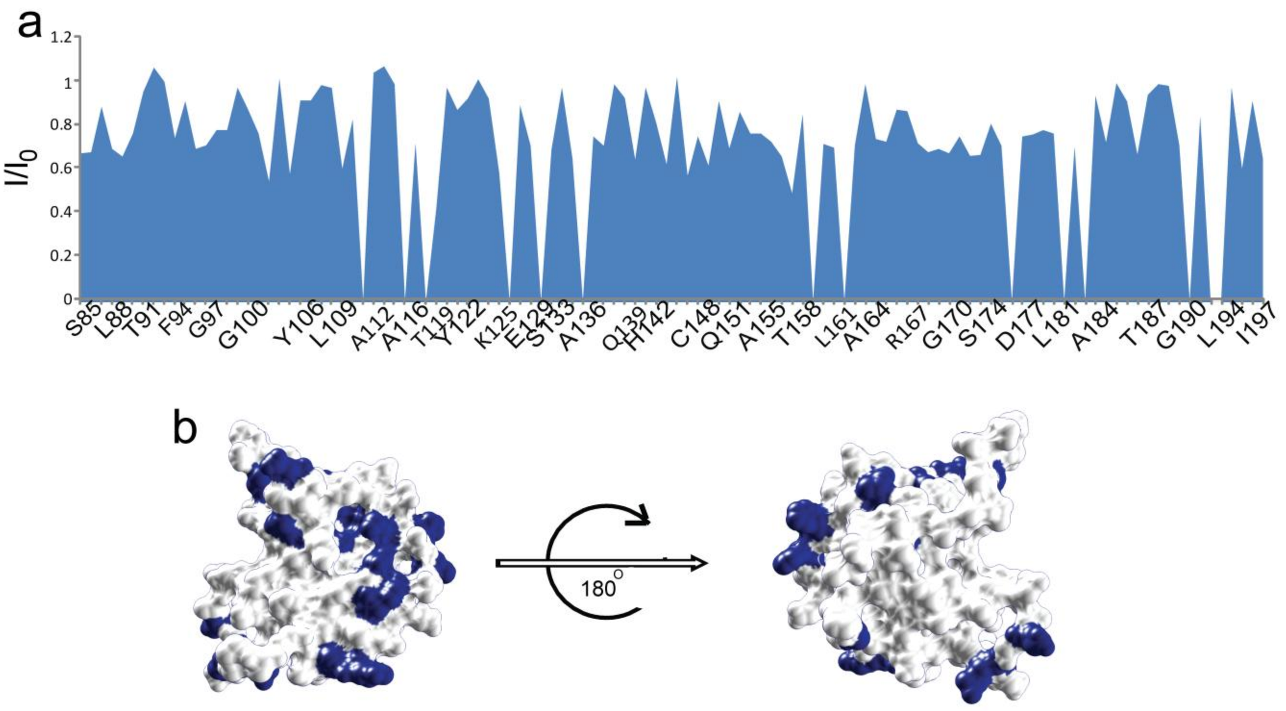

Supplementary Figure 5. Mapping the interaction site of client peptide B on proSP-C BRICHOS Var II. (a) 100 µM proSP-C BRICHOS Var II was incubated in presence of 1 mM substrate peptide B (VLEMGSGSGSKKVVVVVVKK) at 303 K, pH ~6.8 in phosphate buffer. Intensity of different amino acids of proSP-C BRICHOS Var II was monitored in absence (I_0) and presence (I) of peptide B. NMR signal intensity ratios (I/I_0) were determined for each residue by extracting the maximal signal height of the cross-peaks from the respective 2D $[^{15}N,^{1}H]$ NMR spectra. (b) The interaction site between peptide B and proSP-C BRICHOS Var II was mapped onto the structure of proSP-C BRICHOS Var II by plotting ratios below 0.7.

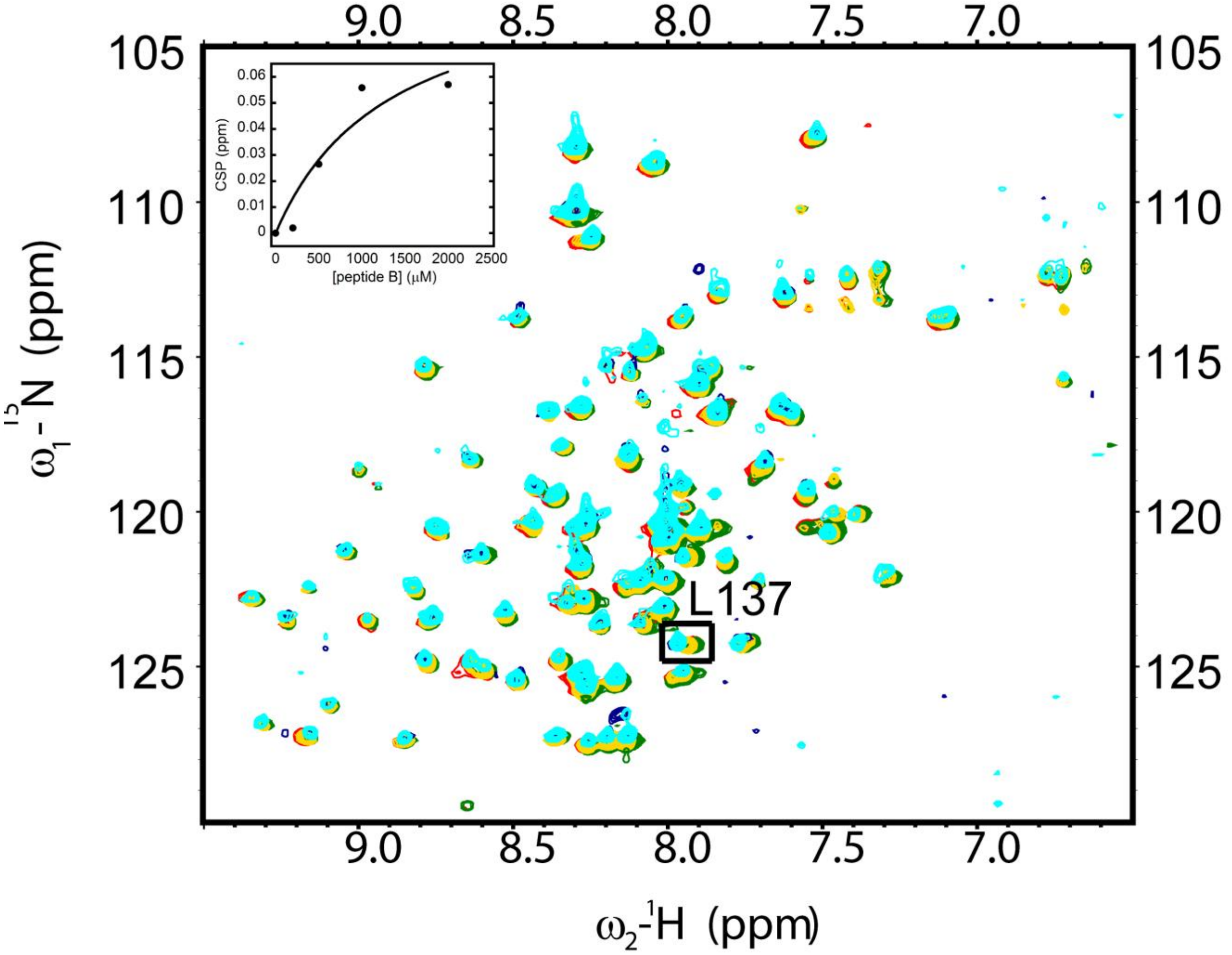

Supplementary Figure 6. **Client peptide B interacts weekly with the proSP-C BRICHOS Var II domain.** 100 µM proSP-C BRICHOS Var II was titrated with increasing concentration of peptide B. Red colour represents proSP-C BRICHOS Var II (100 µM) in absence of any peptide, whereas, green, yellow, cyan and blue represents proSP-C BRICHOS Var II in presence of 200 µM, 500 µM, 1 mM, and 2 mM peptide, respectively. Dissociation constant of peptide B to proSP-C BRICHOS Var II was determined to be ~ 800 µM (inset) suggesting week interactions between peptide B and proSP-C BRICHOS Var II domain. It is stated that the binding affinity could not be determined with high accuracy due to the limitation of the addition of high concentration of peptide B.

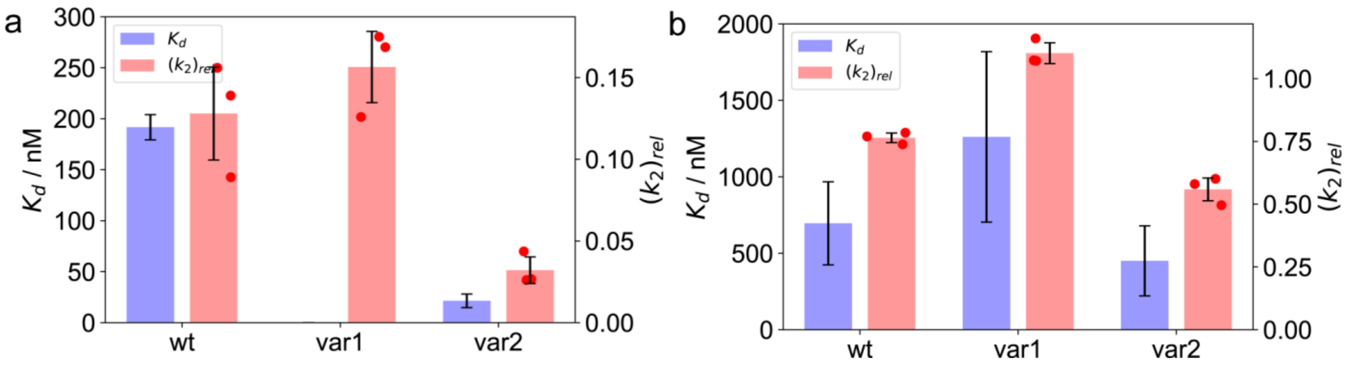

Supplementary Figure 7. Comparison of rate constants and affinities. (a) Comparison of the affinities of the different proSP-C BRICHOS variants against Aβ42 fibrils with the rate constant k_+k_2 at 7.5 μM proSP-C BRICHOS concentration. For proSP-C BRICHOS Var I, no affinity has been detectable. (b) Comparison of the affinities of the different proSP-C BRICHOS variants against α-Syn fibrils with the rate constant k_+k_2 at 10 μM proSP-C BRICHOS concentration. 3 independent experiments were performed for each set and corresponding S.D are represented here.

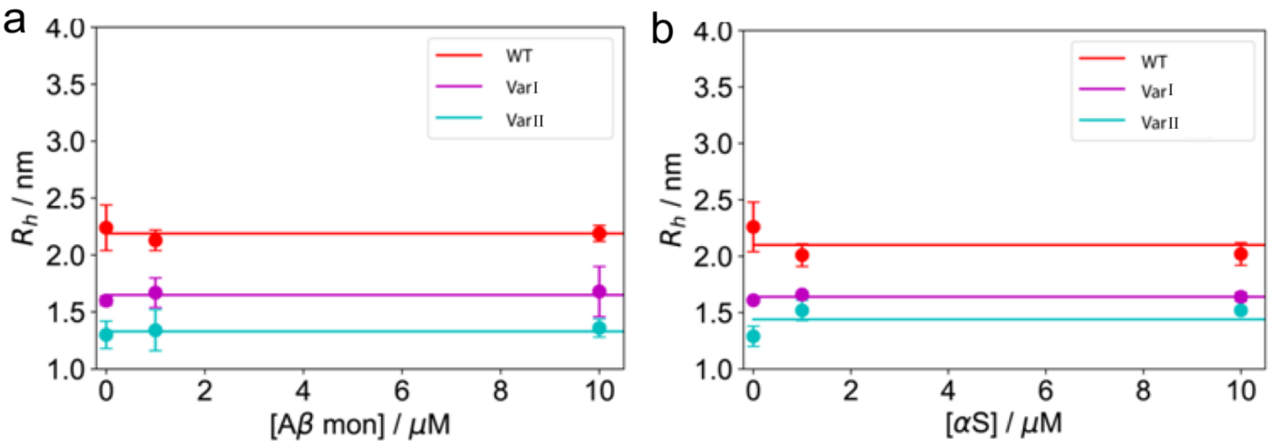

Supplementary Figure 8. Binding experiment of WT proSP-C BRICHOS and its variants to monomeric α-Syn and Aβ42. Microfluidic Diffusional Sizing experiment showing no increment of the hydrodynamic radius of proSP-C BRICHOS upon binding with monomeric Aβ42 (a) and α-Syn (b), which confirms the lack of binding events of proSP-C BRICHOS with monomeric Aβ42 and α-Syn. Each experiment was performed three times and their corresponding S.D is represented.

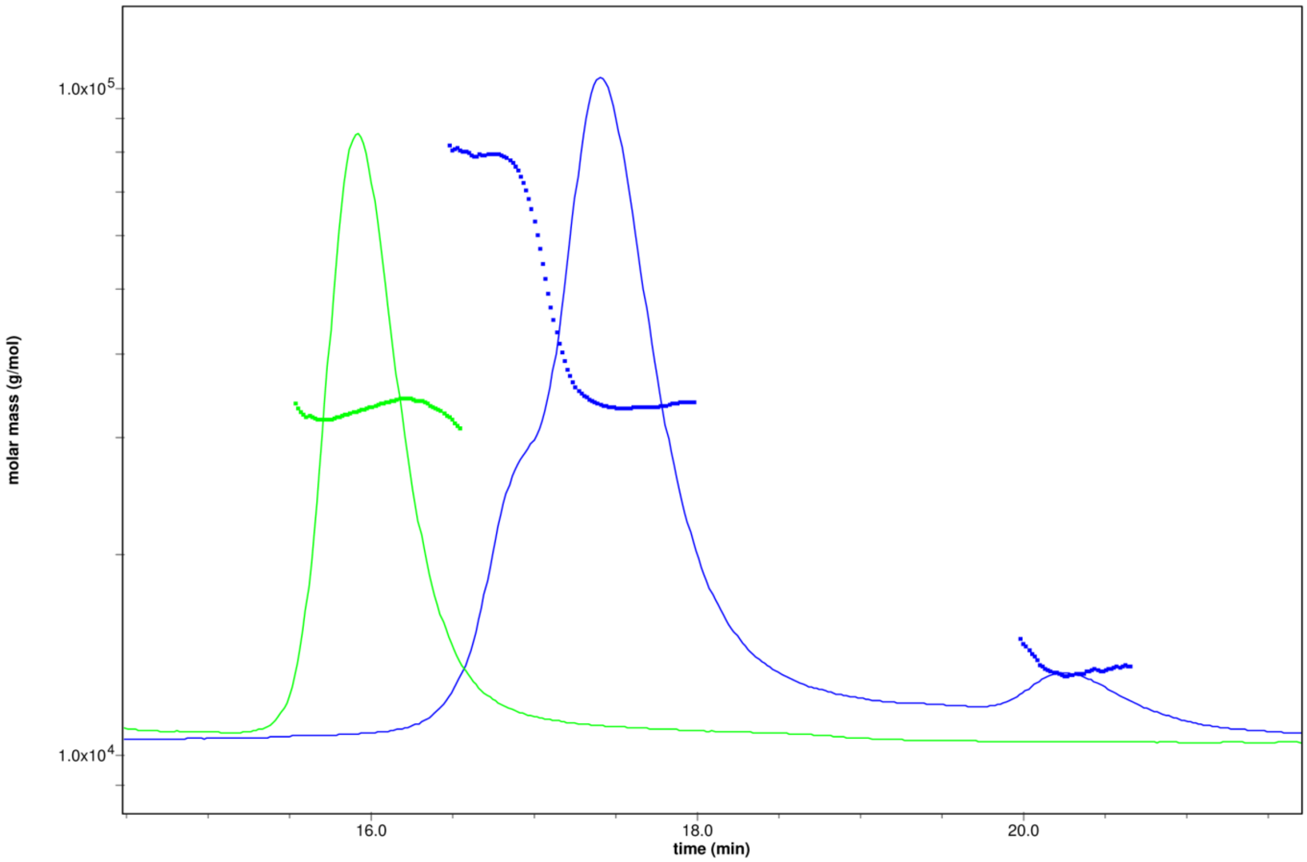

Supplementary Figure 9. Lowering pH causes dissociation of trimeric proSP-C BRICHOS into monomeric entity. The elution profile and molar mass determined by multiangle light scattering of WT proSP-C BRICHOS at pH pH 6.8 (green) demonstrates its trimeric form, while at lower pH 5.8 (blue) it is present both as a trimer and a monomeric entity.

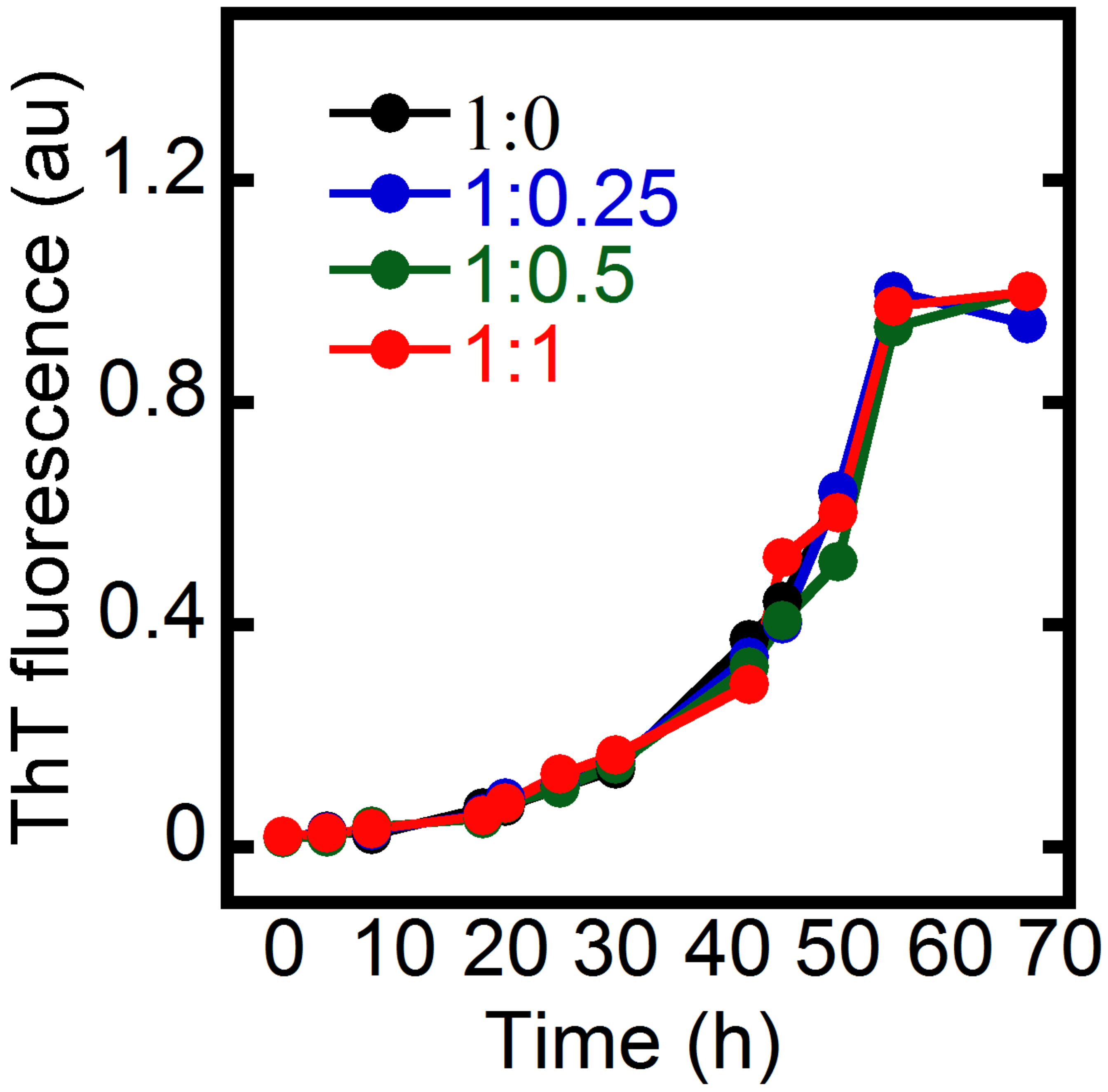

Supplementary Figure 10. proSP-C BRICHOS Var II does not interfere with the aggregation kinetics of C-terminal truncated α-Syn (1-121). Aggregation of α-Syn(1-121) (~150 µM, pH ~7.4, 37 °C in PBS) in presence of varying molar equivalents of proSP-C BRICHOS Var II as indicated (molar ratio of α-Syn: BRICHOS corresponds to 1:0, 1:0.25, 1:0.5 and 1:1 respectively) was monitored by an increase in the fluorescence of thioavin T (ThT). During aggregation, 10 µl sample was collected and diluted in 200 µl PBS and ThT signal was monitored at regular interval. The data indicate that proSP-C BRICHOS Var II does not influence the C-terminal truncated α-Syn aggregation. Two independent experiments were performed for each sample.

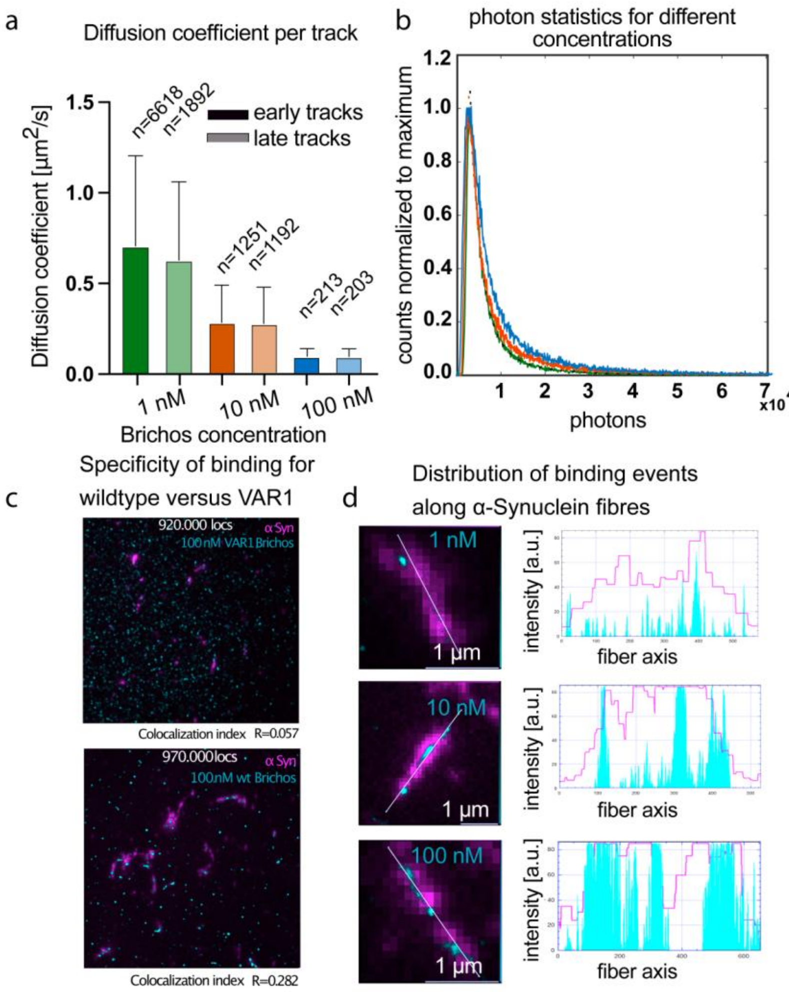

Supplementary Figure 11. Single particle tracking (SPT) of BRICHOS variants along α-Syn fibrils. For further validation of the SPT data, diffusion coefficients were analyzed at different times throughout the experiments, checking if over time proSP-C BRICHOS would fully decorate α-Syn fibrils. In **(A)** diffusion coefficients are shown for tracks originating from the first 10 min of the experiment and second half from 10 – 20 min after labeled proSP-C BRICHOS Var II addition at different concentrations 1- 100 nM. Mean +/- S.D are represented here. **(B)** Photon statistics are plotted, suggesting similar results for different proSP-C BRICHOS Var II concentrations. For SPT analysis, molecules with low photon budget resulting in a localization precision of > 30 nm are excluded. **(C)** For specificity of binding, low affinity proSP-C BRICHOS Var I and WT proSP-C BRICHOS are compared, therefore similar number of localizations is plotted. Colocalization analysis resulted in higher index (via Pearson correlation) for WT versus proSP-C BRICHOS Var I. **(D)** Examples of localization density along α-Syn fibrils are plotted for different WT proSP-C BRICHOS concentrations suggesting an inhomogeneous distribution along the main fibril axis at higher concentration.

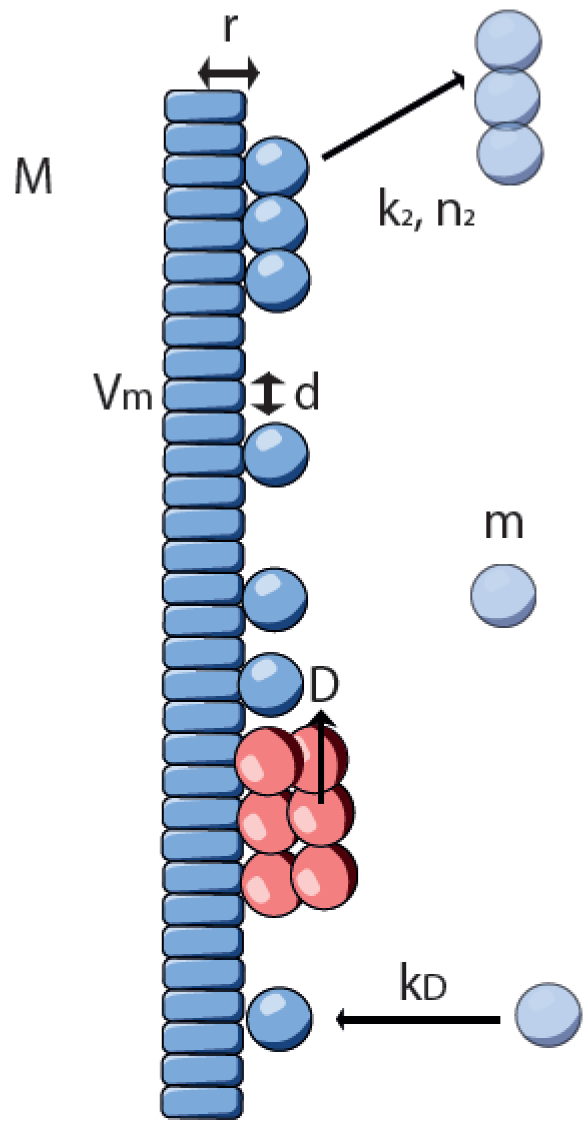

Supplementary Figure 12.Schematic of the kinetic model of proSP-C BRICHOS action in interference with α-Syn secondary nucleation. M is fibrils mass concentration, m is free monomer concentration, r is the radius of cross section of fibrils, d is the periodic repeat distance of intermolecular β -sheets along the fibrils direction, V_m is the volume of one monomer in the fibril, k_2, n_2 are the secondary nucleation rate constant and the secondary nucleation reaction order, D is the diffusion coefficient of the chaperone on the fibril and k_D is the diffusion limited rate constant of monomers adsorbed on the fibril.

Supplementary Table 1 | NMR structure statistics for BRICHOS variant II and trimer

	**Variant II**	**Trimer**
**NMR restraints**		
NOE distance restraints:		
Total	820	2372
Short-range ($\|i-j\| \leq 1$)	638	1385
Medium-range ($2 \leq \|i-j\| \leq 4$)	46	349
Long-range ($\|i-j\| \geq 5$)	136	638
Hydrogen bond restraints	0	0
Dihedral angle restraints (ϕ/ψ)	118	318
**Structure statistics**	Mean ± s.d.	Mean ± s.d.
CYANA target function value ($Å^2$)	7.28 ± 0.33	24.01 ± 1.20
Distance restraint violations > 0.5 Å	1 ± 1	4 ± 3
Dihedral angle restraint violations > 5º	4 ± 2	3 ± 1
Ramachandran plot statistics (PROCHECK):		
Residues in most favored regions (%)	68.9	60.8
Residues in additionally allowed regions (%)	25.1	32.7
Residues in generously allowed regions (%)	4.1	4.7
Residues in disallowed regions (%)	1.9	1.9
RMSD to mean coordinates (residues 91–148, 177–197):		
Backbone N, C^{α}, C' (Å)	1.06 ± 0.19	0.70 ± 0.14
Heavy atoms (Å)	1.64 ± 0.24	1.06 ± 0.14

Supplementary note. Kinetic Model of Secondary Nucleation Interference by proSP- C BRICHOS

A schematic of the kinetic model of secondary nucleation interference of proSP-C BRICHOS is illustrated in Supplementary Figure 12. It is based on a cylindrical fibril with radius r = 5 nm and α-Syn monomer volume V_m = 16 nm^3 derived from the cryo EM structure[1]. Based on the experimentally derived stoichiometry of 1:10 chaperone molecules (proSP-C BRICHOS:α-Syn monomer) adsorbed α-synuclein molecule in the fibril, we obtain an area A = 10*Vm*2/r = 64nm2 that each chaperone needs to sweep.

Following the equation $t=AD^{-1}$ it results $t \approx 6.4*10^{-4}$ s for proSP-C BRICHOS to move through the area A, where D = 0.1µm2/s is the diffusion coefficient of the chaperone on the fibril.

Next the number of secondary nuclei generated in the area A during the time t is calculated using previously reported literature[2] using equation 1. First, we consider the total number of secondary nuclei formed per unit time for a system with a (monomer mass equivalent) concentration M of fibrils (10^{-5}mol/L) and a concentration of monomer of m (10^{-5}mol/L):

$$dN = N_A k_2 m^{n_2} M dt = N_A k_2 m^{n_2} N_{\text{agg}}/N_A dt = k_2 m^{n_2} N_{\text{agg}} dt \qquad (1)$$

where N_{agg} is the number of monomers per unit volume.

Using the cylindrical geometry of the fibril $N_{agg} = \pi r^2 h/V_m$ and the number of monomers per area is $r/2V_m$, the number of secondary nuclei formed in time t on a given surface area A is determined using equation 2.

$$N = k_2 m^{n_2} \frac{r}{2V_m} \cdot A \cdot t \qquad (2)$$

where k_2, n_2 are the secondary nucleation rate constant and the secondary nucleation reaction order, respectively[2-6].

Evaluating for the fibril geometry and A = 64 nm^2, proSP-C BRICHOS diffusion time through the area A t = $6.4*10^{-4}$ s and $k_2 m^{n2} \approx 1.5\cdot10^{-6} s^{-1}$[6,7] yields N ≈ $9.6\cdot10^{-9} \ll 1$. This suggests that proSP-C BRICHOS interferes with secondary nucleation formation by passing a (many) nucleation site(s). With other words, the passing is much faster than the formation of the secondary nucleus. While passing BRICHOS competes with the binding site of α-Syn monomer kicking it off the fibril. Albeit the readsorbtion of α-Syn monomer is on the time scale of microseconds as determined by NMR experiments [8] and by an estimation of a diffusion limited rate constant (see below) and thus many orders of magnitude slower than the secondary nucleation formation. Therefore, by swiping along the fibril surface proSP-C BRICHOS is perturbing the formation of the secondary nucleation.

The readsoprtion time of α-Syn monomer is given by equation 3 and 4.

$$t_{ad} = \frac{0.5\cdot m}{k_D\cdot m\cdot M} = \frac{0.5}{k_D\cdot M} \qquad (3)$$

$$D_{mM} = \frac{k_B T}{6\pi\eta}\left(\frac{1}{R_M} + \frac{1}{R_m}\right) \approx D_m = 100\mu m^2/s \qquad (4)$$

Where $K_D=4\pi D_{mM}..R_{mM}$ is is the diffusion limited rate constant of monomers adsorb on fibrils D_{mM} is coefficient of mutual diffusion of monomers and fibrils and R_{mM} is the encounter distance between monomer and fibril for adsorption happens

Taken together this yields a t_{ad} = 6.6 $*10^{-5}$ s, which is similar to the experimental value.

References.

1 Frey, L. *et al.* On the pH-dependence of α-synuclein amyloid polymorphism and the role of secondary nucleation in seed-based amyloid propagation.

2 Gaspar, R. *et al.* Secondary nucleation of monomers on fibril surface dominates α-synuclein aggregation and provides autocatalytic amyloid amplification. *Quarterly reviews of biophysics* **50**, e6, doi:10.1017/s0033583516000172 (2017).

3 Törnquist, M. *et al.* Secondary nucleation in amyloid formation. *Chemical communications (Cambridge, England)* **54**, 8667-8684, doi:10.1039/c8cc02204f (2018).

4 Hadi Alijanvand, S., Peduzzo, A. & Buell, A. K. Secondary Nucleation and the Conservation of Structural Characteristics of Amyloid Fibril Strains. *Frontiers in molecular biosciences* **8**, 669994, doi:10.3389/fmolb.2021.669994 (2021).

5 Iljina, M. *et al.* Kinetic model of the aggregation of alpha-synuclein provides insights into prion-like spreading. *Proceedings of the National Academy of Sciences of the United States of America* **113**, E1206-1215, doi:10.1073/pnas.1524128113 (2016).

6 Catherine, K. X. *et al.* α-Synuclein oligomers form by secondary nucleation. *bioRxiv*, 2023.2005.2028.542651, doi:10.1101/2023.05.28.542651 (2024).

7 Buell, A. K. *et al.* Solution conditions determine the relative importance of nucleation and growth processes in α-synuclein aggregation. *Proceedings of the National Academy of Sciences of the United States of America* **111**, 7671-7676, doi:10.1073/pnas.1315346111 (2014).

8 Kumari, P. *et al.* Structural insights into α-synuclein monomer-fibril interactions. *Proceedings of the National Academy of Sciences of the United States of America* **118**, doi:10.1073/pnas.2012171118 (2021).

.
