# Supplementary material for: The inhibitory action of the chaperone BRICHOS against the α-Synuclein secondary nucleation pathway
Source: Nat Commun. 2024 Nov 20;15:10038. doi: 10.1038/s41467-024-54212-2 (PMC11579453; doi:10.1038/s41467-024-54212-2)
Supplement: Supplementary file 3 — Description of Additional Supplementary Files [file 41467_2024_54212_MOESM3_ESM.pdf]

Description of Additional Supplementary Files

File name: Supplementary Movie 1

Description: proSP-C BRICHOS Var II diffuses along the α-Syn fibrils.

Interplay between proSP-C BRICHOS Var II and α-Syn amyloid fibrils was monitored by time-resolved super-resolution fluorescence microscopy where proSP-C BRICHOS Var II was fluorescently labeled with Atto64 dye. Total internal reflection fluorescence microscopy reveals 1D diffusion of proSP-C BRICHOS Var II along α-Syn fibrils.
